# Effects of Oligosaccharides From *Morinda officinalis* on Gut Microbiota and Metabolome of APP/PS1 Transgenic Mice

**DOI:** 10.3389/fneur.2018.00412

**Published:** 2018-06-15

**Authors:** Yang Xin, Chen Diling, Yang Jian, Liu Ting, Hu Guoyan, Liang Hualun, Tang Xiaocui, Lai Guoxiao, Shuai Ou, Zheng Chaoqun, Zhao Jun, Xie Yizhen

**Affiliations:** ^1^Department of Pharmacy, The Fifth Affiliated Hospital of Guangzhou Medical University, Guangzhou, China; ^2^The Fifth Clinical School of Guangzhou Medical University, Guangzhou, China; ^3^State Key Laboratory of Applied Microbiology Southern China, Guangdong Provincial Key Laboratory of Microbial Culture Collection and Application, Guangdong Open Laboratory of Applied Microbiology, Guangdong Institute of Microbiology, Guangzhou, China; ^4^Department of Pharmacy, The Second Clinical Medical College of Guangzhou University of Chinese Medicine, Guangzhou, China; ^5^College of Pharmacy, Guangxi University of Traditional Chinese Medicine, Nanning, China; ^6^Department of Obstetrics, Guangdong Women and Children Hospital, Guangzhou, China

**Keywords:** oligosaccharides of *Morinda officinalis*, Alzheimer's disease, gut microbiota, metabolomics, APP/PS1 transgenic mice, metabolites

## Abstract

Alzheimer's disease (AD), a progressive neurodegenerative disorder, lacks preclinical diagnostic biomarkers and therapeutic drugs. Thus, earlier intervention in AD is a top priority. Studies have shown that the gut microbiota influences central nervous system disorders and that prebiotics can improve the cognition of hosts with AD, but these effects are not well understood. Preliminary research has shown that oligosaccharides from *Morinda officinalis* (OMO) are a useful prebiotic and cause substantial memory improvements in animal models of AD; however, the mechanism is still unclear. Therefore, this study was conducted to investigate whether OMO are clinically effective in alleviating AD by improving gut microbiota. OMO were administered to APP/PS1 transgenic mice, and potential clinical biomarkers of AD were identified with metabolomics and bioinformatics. Behavioral experiments demonstrated that OMO significantly ameliorated the memory of the AD animal model. Histological changes indicated that OMO ameliorated brain tissue swelling and neuronal apoptosis and downregulated the expression of the intracellular AD marker Aβ_1−42_. 16S rRNA sequencing analyses indicated that OMO maintained the diversity and stability of the microbial community. The data also indicated that OMO are an efficacious prebiotic in an animal model of AD, regulating the composition and metabolism of the gut microbiota. A serum metabolomics assay was performed using UHPLC-LTQ Orbitrap mass spectrometry to delineate the metabolic changes and potential early biomarkers in APP/PS1 transgenic mice. Multivariate statistical analysis showed that 14 metabolites were significantly upregulated, and 8 metabolites were downregulated in the model animals compared to the normal controls. Thus, key metabolites represent early indicators of the development of AD. Overall, we report a drug and signaling pathway with therapeutic potential, including proteins associated with cognitive deficits in normal mice or gene mutations that cause AD.

## Introduction

As an irreversible neurodegenerative disease that causes cognitive deficits, Alzheimer's disease (AD) accounts for 60–80% of all types of dementia (1). The typical neuropathological features of AD include the extracellular aggregation of amyloid-β (Aβ) peptide, the formation of tau protein aggregates inside neurons and the malfunction and/or loss of synapses and axons (2–4). Since ~14 million people in the United States are predicted to be diagnosed with AD by 2050 (5), investigations of agents and reactions whose dysfunction causes AD are essential to provide insights into the etiology of the illness and to develop effective treatment strategies.

Some pathological features of AD, including cerebral atrophy, amyloid generation, altered gene expression, altered immune reactions, and cognitive deficits, have recently been linked to microbe infections (6–10). The brain and gut interact and modulate each other (11), and microbes in the gastrointestinal tract (GIT) are postulated to participate in AD development (12–14), although our understanding of the development and pathology of AD is insufficient and changing. Researchers have not determined how an imbalance in GIT microbes affects AD. The impact may be associated with the invasion of several pathogens, a decrease in the number of protective microbes, impaired immune tolerance, elevated membrane permeability and other defects in the immune system (15, 16). Probiotics have been shown to participate in the ability of the host to prevent and manage both chronic and acute conditions, such as AD (17). Alterations in the host physiology resulting from aging, genetics, diet, lifestyle and other factors might noticeably affect microbes (18–21). Metabolomics is a postgenomic field that offers new insights into the diagnostic and prognostic biomarkers of AD (22). Metabolomics has been used to identify metabolites and determine the expression of related genes in whole organisms, and it has recently been used in a wide range of applications in the clinic. Serum metabolites were discovered and considered potential biomarkers of AD (23), as retinoate has been used to help characterize and discriminate pathophysiological signatures of AD (24). According to recent reports, the gut microbiota and their metabolites influence the host metabolism (25). However, the roles of the gut microbiota and serum metabolites in AD are unclear.

Oligosaccharides from *Morinda officinalis* (OMO), a natural herbal extract used in traditional Chinese medicine, contain many active components. The saccharide content in the *M. officinalis* root is as high as 49.8–58.3%, mainly consisting of oligosaccharides with anti-dementia and memory-enhancing effects on many animal models (26–28). However, the mechanism underlying these beneficial effects has not been identified and will be elucidated in the present study. This study aims to provide a basis for the effective prediction and characterization of AD pathology by analyzing the gut microbiota and serum metabolite biomarkers.

Our research utilizes transgenic APPswe/PS1dE9 (APP/PS1) mouse models (29), which have been widely applied to investigations of the initial etiology of AD and evaluations of the efficacy of OMO treatments. Every specimen containing microbial 16S rRNA genes underwent concentration with the help of solid-phase reversible immobilization (SPRI) and subsequent quantification by electrophoresis utilizing an Agilent 2100 Bioanalyzer (Agilent, USA) prior to sequencing on an Illumina MiSeq sequencing system (30).

Whole-body blood specimens acquired from 8-month-old APP/PS1 and C57 mice were subjected to metabolomics evaluations using UHPLC-MS/MS. Subsequently, the entirety of the spectra were assessed using multivariate analysis to holistically investigate alterations in the levels of circulating metabolites. Fourteen metabolites were recognized that might contribute to the diagnosis and treatment of AD in the initial stage. Consequently, our research focused on assessing the therapeutic impact of OMO on AD by analyzing the diversity of microbes and performing metabolomics assays to provide insights into its etiology. Moreover, the results from this study will serve as the basis for the application of nutritional interventions and the AD-counteracting effects of OMO.

## Methods

### Animal model preparation and treatments

Adult male C57 mice and 2-month-old transgenic APP/PS1 mice weighing 18 to 22 grams were acquired from the Laboratory Animal Center of Guangdong Province (SCXK [Yue] 2008-0020, SYXK [Yue] 2008-0085) and were housed in pairs in the colony room on a 12/12-h light/dark cycle at 25°C in plastic cages and were allowed *ad libitum* access to food and water. Each procedure described in our study was approved by the Laboratory Animal Center of the Guangdong Institute of Microbiology. This study utilized as few mice as possible. 2-month-old APP/PS1 mice and age-matched C57 mice, which served as the control group (*n* = 10 animals), were utilized in the present study. Our research was approved by the Ethical Committee and complied with the Declaration of Helsinki.

Mice performed water maze tests (WMT) to identify adequate AD models before animals were randomized into the following 4 groups in this 6-month experiment: a C57 group (oral administration of distilled water), a transgenic APP/PS1 group [oral administration of distilled water], a high-dose transgenic APP/PS1 group [oral administration of 100 mg/(kg·d) OMO] and a low-dose transgenic APP/PS1 group (oral administration of 50 mg/(kg·d) OMO) (*n* = 10 animals per group).

### WMTs

A Morris water maze (MWM, DMS-2, Chinese Academy of Medical Sciences Institute of Medicine) comprising a non-transparent circular fiberglass pool with a diameter of 20 cm that had been filled with water (25 ± 1°C) was used to examine murine spatial learning and memory. Wathet curtains tagged with three distal visual cues encircled the pool. Unified lighting of the examination room and pool was provided by four independent light sources of equivalent power. A CCD camera was placed over the center of the pool to record the swimming paths of each mouse. An EthoVision tracking system (Noldus, Leesburg, VA) was applied to digitize the video output. The WMT consisted of three steps, as described in a previous study (31): primary spatial practice, reverse spatial practice and a probe examination.

### Hematoxylin-eosin (HE) and immunohistochemical staining

The look, activity and fur tint of each mouse were examined and recorded daily. The weight of each mouse was examined every 3 days when drugs were administered. Serum specimens were harvested, and murine cerebral samples were anatomically dissected after the WMT.

Three cerebral samples and three intestinum tenues were removed from randomly chosen mice in each experimental group, while the remaining tissues underwent fixation with four percent paraformaldehyde before processing into paraffin sections. Sections were stained with HE and immunohistochemistry before being examined under the light microscope (31, 32).

### Microbiome analysis

Fresh fecal specimens were acquired from murine nests and stored at −80°C. Microbe DNA specimens weighing from 1.2 to 20.0 ng were isolated from murine cecal specimens and stored at −20°C. The 16S rRNA genes of microbes were amplified with the following primers: forward 5′-ACTCCTACGGGAGGCAGCA-3′ and reverse 5′-GGACTACHVGGGTWTCTAAT-3′. Every amplified product was concentrated by SPRI and quantified by electrophoresis using an Agilent 2100 Bioanalyzer (Agilent). Every specimen was diluted to 1 × 10^9^ molecules/μL in TE buffer and pooled into groups prior to the determination of DNA concentrations using a NanoDrop spectrophotometer. An Illumina MiSeq sequencing system was utilized to sequence 20 mL of the pooled admixture. The subsequent reads were analyzed as described in a previous study (30).

### Serum metabolomics analysis

Eighty microliters of mouse serum was combined with 240 μL of a cold methanol-acetonitrile (2:1, v/v) mixture and 10 μL of an internal standard (0.3 mg/mL 2-chloro-L-phenylalanine in methanol). After vortexing for 2 min, the mixture was subjected to ultrasound disruption for 5 min, incubated in a −20°C freezer for 20 min, and centrifuged for 10 min at a low temperature (14,000 rpm at 4°C). Subsequently, 200 μL of the supernatant was loaded into the tube lining a sample vial and analyzed using LC-MS (33).

#### LC-MS analysis using an UHPLC-LTQ orbitrap spectrometer

An Ultimate 3000-Velos Pro system was utilized for LC-MS with the help of a binary solvent delivery manager and a specimen manager connected to an LTQ Orbitrap mass spectrometer equipped with an electrospray interface (Thermo Fisher Scientific, USA). LC settings are displayed below. The Acquity BEH C18 column (100 mm × 2.1 mm internal diameter, 1.7 μm; Waters, Milford, USA) was stored at 45°C. Isolation was performed with the following solvent gradient: 5% B−25% B from 0 to 1.5 min, 25% B−100% B from 1.5 to 10.0 min, 100% B−100% B from 10.0 to 13.0 min; 100% B−5% B from 13.0 to 13.5 min, and 13.5–14.5 min holding at 5% B at a flow rate of 0.40 mL/min. B represents acetonitrile (0.1% (v/v) acetonitrile), while A represents aqueous formic acid (0.1% (v/v) formic acid). The injection volume was 3 mL, and the column temperature was set to 45.0°C. Mass spectrometry data were recorded by the LTQ Orbitrap mass spectrometer in negative or positive electrospray ionization (ESI) mode. The capillary and source temperatures were 350°C, and the desolvation gas flow was maintained at 45 L/h. Centroid data were acquired from fifty to 1,000 m/z, with a 30,000 resolution.

#### Multivariate statistical analysis and metabolite identification

The XCMS program (https://xcmsonline.scripps.edu/landing_page.php?pgcontent=mainPage) was used for the non-linear alignment of data in the time domain and the spontaneous integration and isolation of peak intensities. The subsequent 3D matrix, including data such as retention time and m/z pairs (variable indices), specimen names (observations) and normalized ion intensities (variables), were integrated into the SIMCA program (version 14.0, Umetrics, Umeå, Sweden), in which a partial least squares discriminant analysis (PLS-DA), orthogonal partial least squares discriminant analysis (OPLS-DA) and principal component analysis (PCA) were conducted. Model quality was assessed using R^2^X or R^2^Y as well as Q^2^-values. R^2^X or R^2^Y represent the percentage of variance in data interpreted using different models and suggested the goodness of fit, while Q^2^ represented the prediction from the models detected using the cross-validation procedure. As a default, 7 rounds of cross-validation were conducted using SIMCA throughout the experiment to identify the most appropriate quantity of essential ingredients and prevent excessive model fitting. A permutation evaluation (200 times) was performed to confirm the OPLS-DA results.

#### Metabolomic pathway analysis

The web-based instrument Metabolomic Pathway Analysis (MetPA) (http://www.metaboanalyst.ca/faces/ModuleView.xhtml) was used to build and observe the metabolic pathways influenced by OMO by collecting information from databases such as the Kyoto Encyclopedia of Genes and Genomes (KEGG) and Human Metabolome Database (HMDB). The evaluation of KEGG pathways required Goatools (https://github.com/tanghaibao/Goatools) and KEGG (https://www.kegg.jp/) (34–37).

### Statistical analysis

The results are presented as the means ± SD from at least 3 independent experiments. ANOVA was employed to examine the significance of differences between groups using the Statistical Package for the Social Sciences software (SPSS, Abacus Concepts, Berkeley, CA) and Prism 5 (GraphPad Software, San Diego, USA) software. *P* < 0.05 indicated a significant difference.

## Results

### OMO administration improved the AD parameters in APP/PS1 transgenic mice

**Figure 1:**
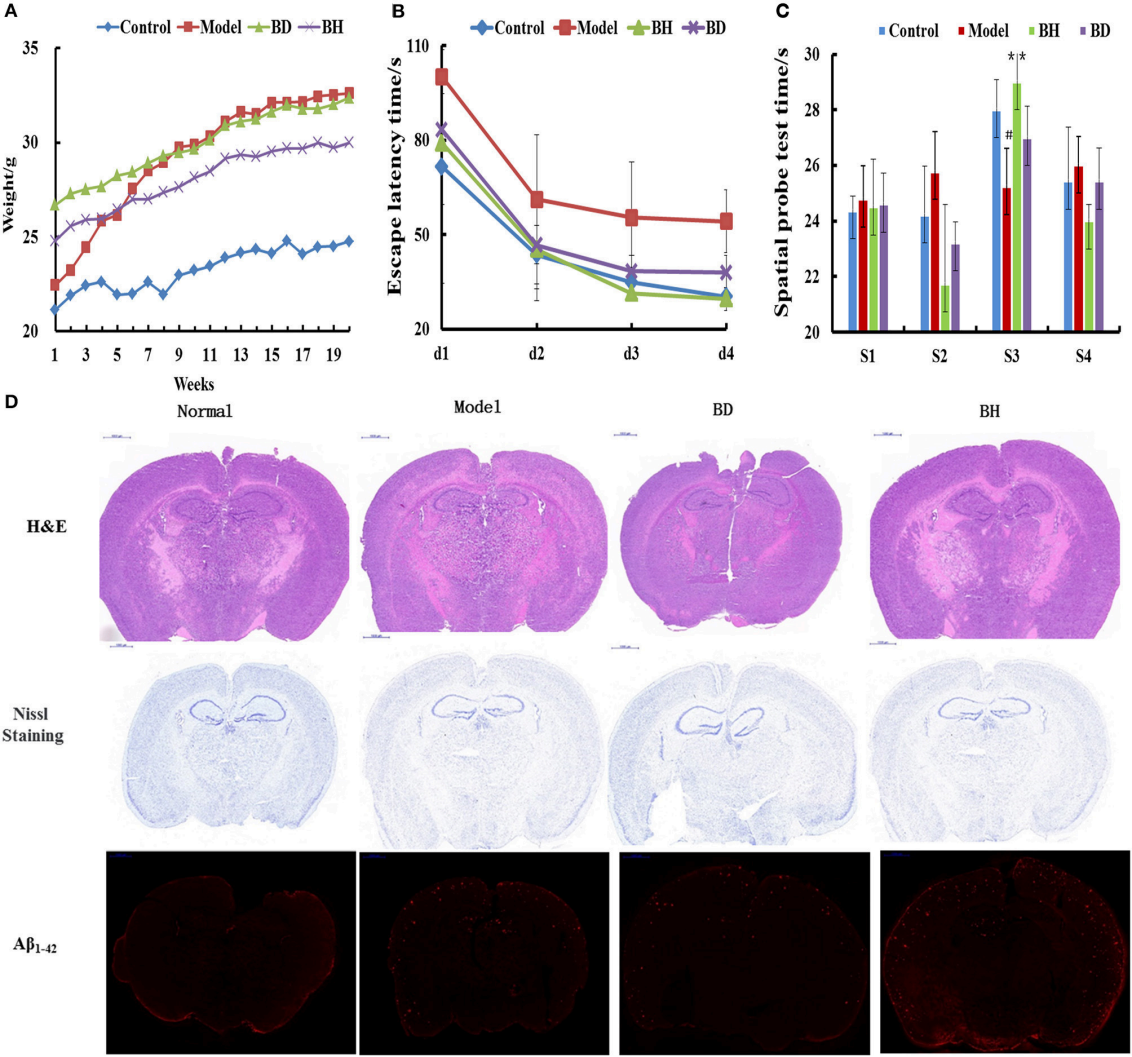
Effect of OMO on APP/PS1 transgenic mice. Body weight changes were measured weekly **(A)**. Escape latencies in the H maze **(B)** and probe test results **(C)**, and histopathological changes in brain tissues **(D)** are shown. N represents the C57 group, M denotes the APP/PS1 transgenic group, BD indicates the group treated with 50 mg/kg OMO, and BH designates the group treated with 100 mg/kg OMO; the treatments were administered for 6 months (*n* = 10). Values are presented as the means ± SDs of six independent experiments. ^#^*p* < 0.05 compared with the control group; ^**^p < 0.01 compared with the M group.

The average weight of the APP/PS1 group was increased compared with that of the control group (*p* < 0.05), and the former group exhibited abdominal swelling. The mice treated with OMO exhibited insignificant differences in weight compared with the control group (*p* > 0.05, Figure 1A).

The incubation time (IT) of every group treated with OMO was noticeably shorter than that of the APP/PS1 group. Mice that received a low dose of OMO displayed an IT of 86.49 ± 11.64 s, while mice receiving the high dose exhibited an IT of 82.06 ± 19.44 s on the 1st day; both values were noticeably different (*p* < 0.01) from the model group (113.75 ± 16.11). On the 4th day, the IT of mice treated with a low dose of OMO was reduced to 37.19 ± 5.36 s, while the IT of the high-dose group decreased to 28.27 ± 3.96 s, both of which differed remarkably from APP/PS1 mice (56.29 ± 9.69 s, *p* < 0.01, Figure 1B). Based on these findings, OMO reversed the learning and memory impairments observed in transgenic APP/PS1 mice.

According to the results of the probe trial, the differences in swimming distance and velocity were insignificant (*p* > 0.05). Mice in the control group swam for longer times in the northwest (target) quadrant (26.63 ± 3.83 s) than in the other quadrants (*p* < 0.01), while mice in the model group displayed a noticeably shorter swimming time (ST) of 20.77 ± 2.36 s (*p* < 0.01) in the aforementioned quadrant, indicating that mice in the control group recalled the position of the platform. The ST of mice receiving a low dose of OMO was 26.50 ± 3.59 s, while the ST of mice receiving the high dose was 27.36 ± 2.51 s, which was noticeably decreased compared with that of the control group. Mice receiving OMO exhibited noticeable differences in ST (*p* < 0.01) from mice in the control group, but the difference in swimming distance was not significant (Figure 1C).

HE staining did not reveal obvious alterations in hippocampal neurons in the control (Figure 1D). However, noticeable histopathological injury was observed in the hippocampus of APP/PS1 mice. Layered pyramidal neuron structure degeneration and neuronal loss (Nissl staining) were observed in the cortex and CA1 area. These alterations were ameliorated by the OMO supplement. Cells in mice receiving OMO displayed a better morphology, and the number of neurons was increased compared with that in mice that were not treated with OMO, particularly in mice receiving the high dose of OMO. The percentage of Aβ_1−42_-positive cells exhibiting red IHC staining was substantially increased in the model group compared with that in the control group (*p* < 0.05). However, OMO administration decreased Aβ_1−42_ expression (Figure 1D).

### OMO administration improved the gut microbiota in APP/PS1 transgenic mice

**Figure 2:**
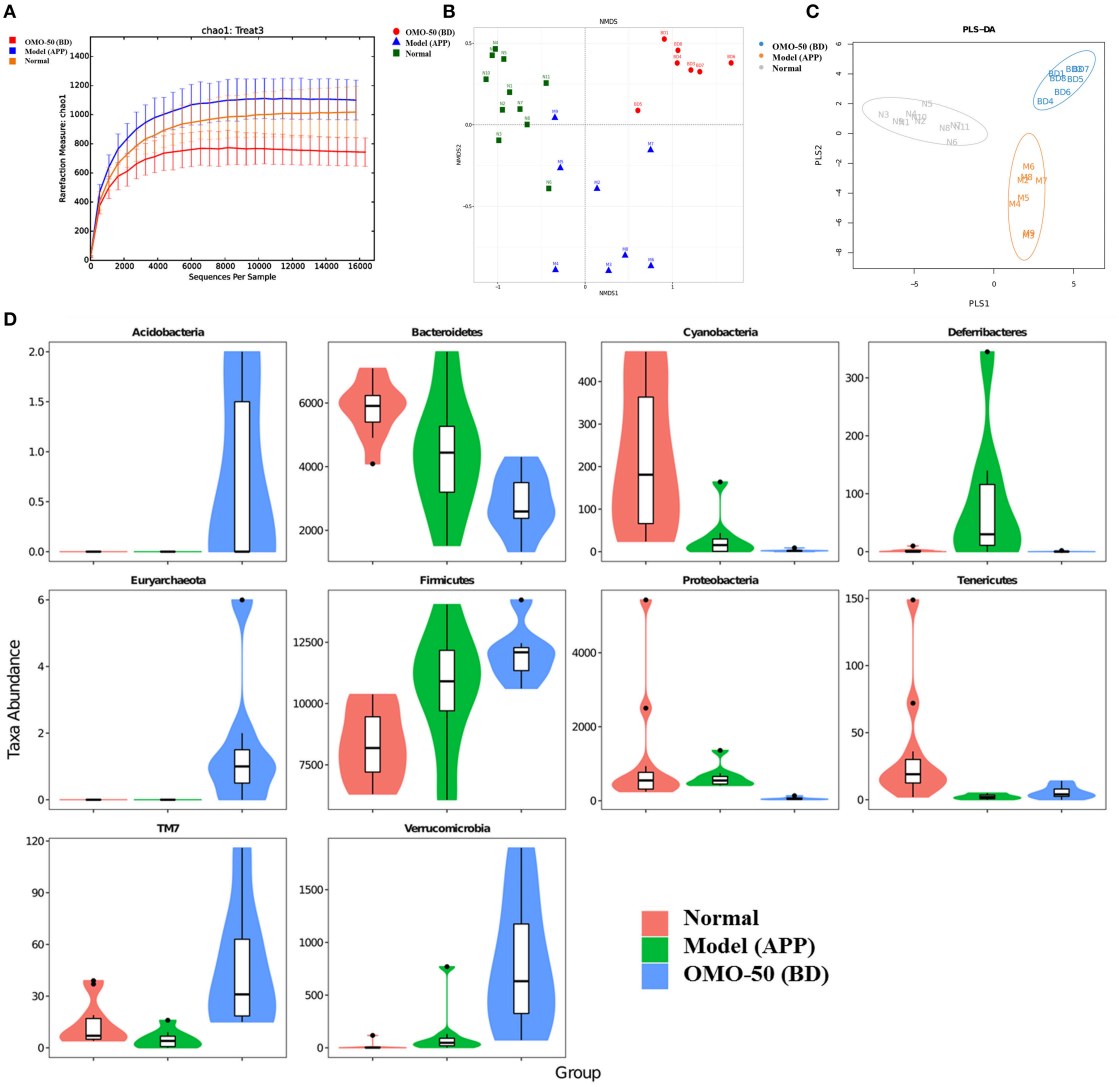
Effects of OMO on the gut microbiota of the APP/PS1 transgenic mice, as determined from fecal samples. The rarefaction curve **(A)**, the results of the NMDS analysis **(B)**, PLS-DA results **(C)**, classification and abundance of fecal contents at the phylum level **(D)**, and the results of 16S rRNA sequencing of the gut microbiota using an Illumina MiSeq sequencing system are shown. N denotes the C57 group, M represents the APP/PS1 transgenic group, BD denotes the 50 mg/kg OMO-treated group, and BH indicates the 100 mg/kg OMO-treated group.

The abundance of operational taxonomic units (OTUs) and the taxonomic profiles were evaluated (Figure 2).

Compared with the normal mice, the APP/PS1 transgenic mice exhibited a decrease in the microbial community diversity, as shown in Figure 2A, and all the APP/PS1 transgenic mice were clustered well using non-metric multidimensional scaling (NMDS) and PLS-DA (Figures 2B,C), indicating that the APP/PS1 transgenic mouse brain influenced the gut microbiota. After treatment with 50 mg/(kg·d) OMO, microbial diversity was improved to approximately the level of the normal control mice (Figure 2B).

Analysis revealed differences in the abundance of taxa between different groups, as displayed in Figure 2D. The abundance of some bacteria exhibited substantial changes at the genus and family levels in fecal samples from the APP/PS1 mice, while the OMO treatment changed those variations; in particular, obvious increases in the abundance of *Lactobacillus, Allobaculum, Lactobacillaceae*, and *Lachnospiraceae* were observed, indicating that OMO had a prebiotic role in protecting against intestinal dysbacteriosis in the AD model animals.

Moreover, at the genus level, the APP/PS1 transgenic mice exhibited an enrichment of some potential anti-AD microbes, such as *Lactobacillus, Akkermansia, Bacteroides, Adlercreutzia*, and *Desulfovibrio*, and reduced levels of other potential anti-AD microbes, such as *Ruminococcus, Bifidobacterium, Blautia, Oscillospira, Coprococcus, Sutterella*, and *Clostridium*, compared with the normal group (**Figure 4A**). At the family level, the APP/PS1 mice showed an enrichment of some potential anti-AD microbes, such as Lactobacillaceae, Lachnospiraceae, Bacteroidaceae, and Verrucomicrobiaceae, and reduced levels of other potential anti-AD microbes, such as S24–7, Ruminococcaceae, Coriobacteriaceae, Erysipelotrichaceae, and Bifidobacteriaceae, compared with the normal group (**Figure 4B**). Nevertheless, model mice treated with OMO displayed alterations in the quantity of microbes that counteracted AD. Based on these findings, OMO represents a promising treatment to modulate the community structure of GIT microbes.

**Figure 3:**
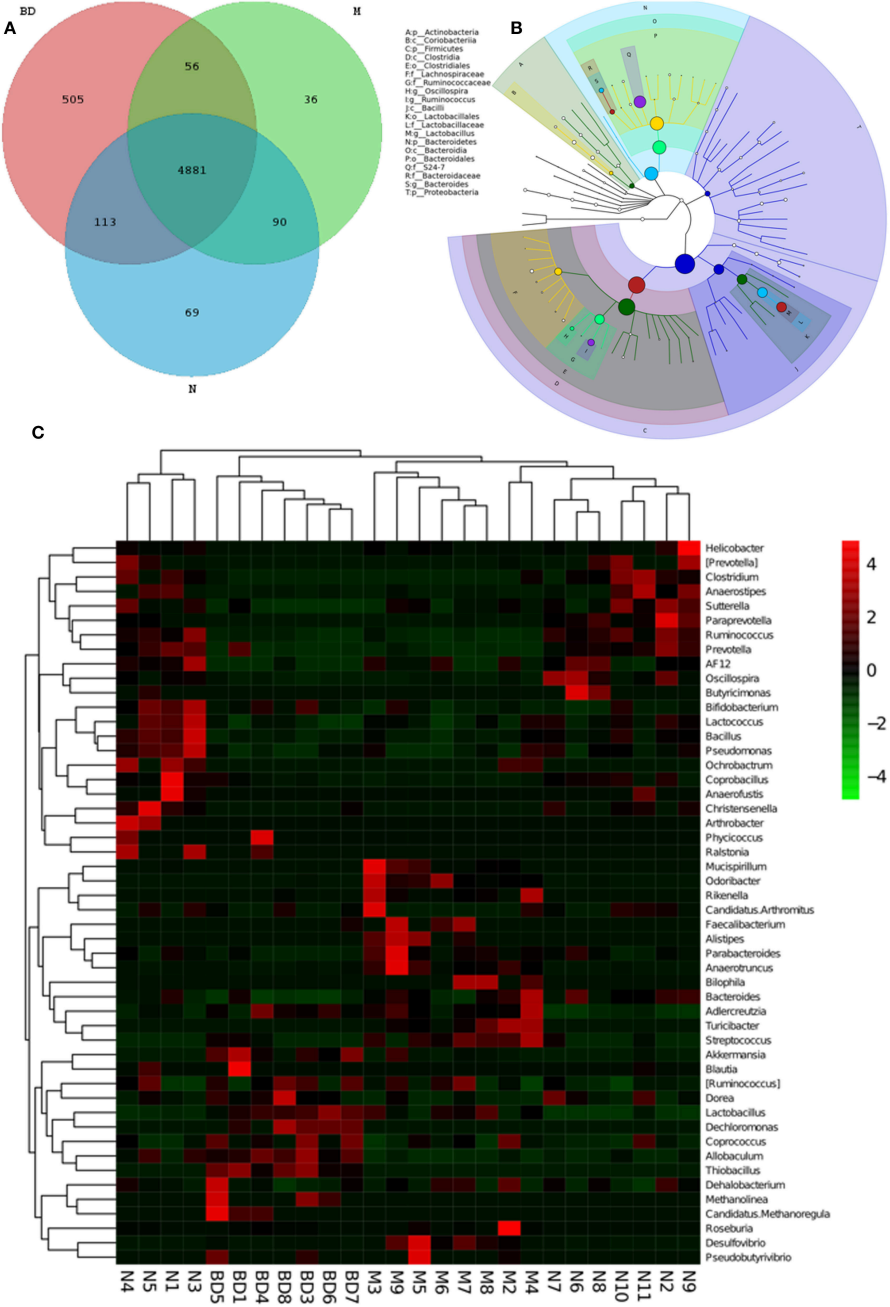
Effects of OMO on the microbiota in fecal samples from the APP/PS1 transgenic mice. A Venn diagram of OTUs **(A)**, a sample species classification tree **(B)**, and the heatmap of 16S rRNA gene sequencing analysis of fecal contents at the genus level **(C)** are shown. N denotes the control group, M denotes the APP/PS1 transgenic group, and BD designates the 50 mg/kg OMO-treated group.

GIT microbes have an obvious impact on the immune system of organisms. The differences in the gut microbiota among the APP/PS1 transgenic, OMO-treated and control mice are shown in Figure 3. A Venn diagram (Figure 3A) and a hierarchical tree (Figure 3B) revealed that Actinobacteria, Firmicutes, Coriobacterium, Lachnospiraceae, Bacilli, Clostridia, Bacteroidales, Clostridiales, Ruminococcaceae, Lactobacillales, Oscillospira, Bacteroidia, and Proteobacteria were the microbial types exhibiting increased levels after OMO administration and are thus promising candidates for future investigation. As shown in the heatmap (Figure 3C), OMO administration remarkably altered the composition of GIT microbes compared to the control group, as the relative abundance levels of the genera *Mucispirillum, Odoribacter, Rikenella, Faecalibacterium, Alistipes, Parabacteroides*, and *Anaerotruncus* were reduced, while the levels of *Arthrobacter, Phycicoccus, Streptococcus, Akkermansia, Blautia, Ruminococcus, Coprococcus, Allobaculum, Dehalobacterium, Methanolinea*, and *Candidatus Methanoregula* were increased. These findings indicate a relationship between the gut microbiota and AD.

**Figure 4:**
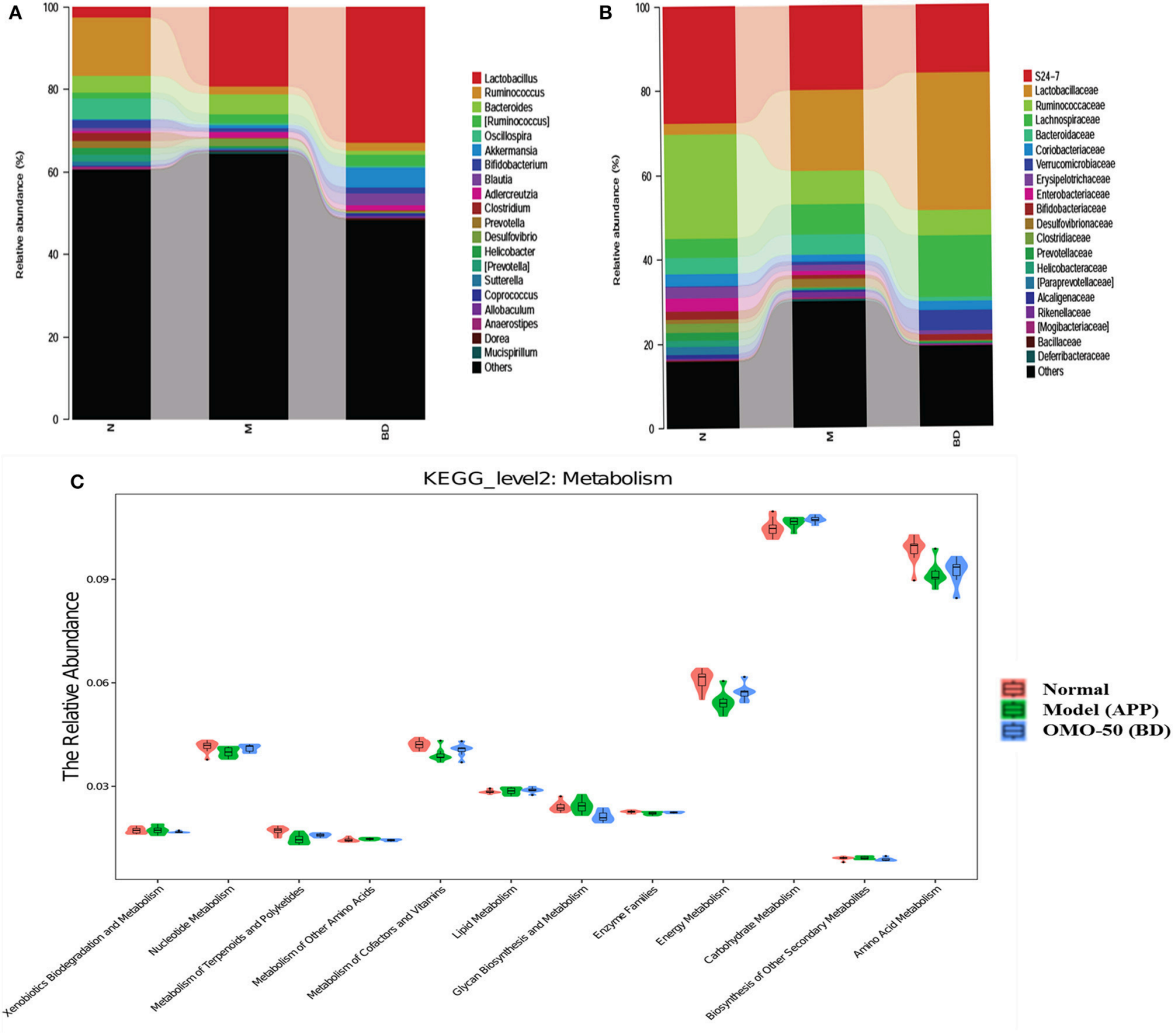
Effects of OMO on the microbiota in fecal samples from the APP/PS1 transgenic mice. The graph in **(A)** shows the classification and abundance of fecal contents at the phylum level, **(B)** shows the classification and abundance of fecal contents at the family level, and **(C)** shows the results of the KEGG pathway enrichment analysis of the gut microbiota with respect to the metabolic systems. Values are presented as the means of six independent experiments. N denotes the control group, M represents the APP/PS1 transgenic group, and BD designates the 50 mg/kg OMO-treated group.

According to the results of the KEGG pathway evaluation, categories of metabolic pathways such as the degeneration of xenobiotics, the generation and metabolism of glycan, the generation of secondary metabolic products, the enzyme families and metabolism of nucleotides, cofactors, terpenoids, amino acids, vitamins, polyketides, lipids, carbohydrate, and energy were altered in GIT microbes of APP/PS1 transgenic animals. Moreover, quite a few of these pathways were upregulated by the OMO supplement. Dynamic alterations in the 3 groups are displayed in Figure 4C, and the results suggested that OMO impacted the GIT microbial metabolism.

### OMO administration changes on metabolites in APP/PS1 transgenic mice

#### Multivariate statistical analysis

**Figure 5:**
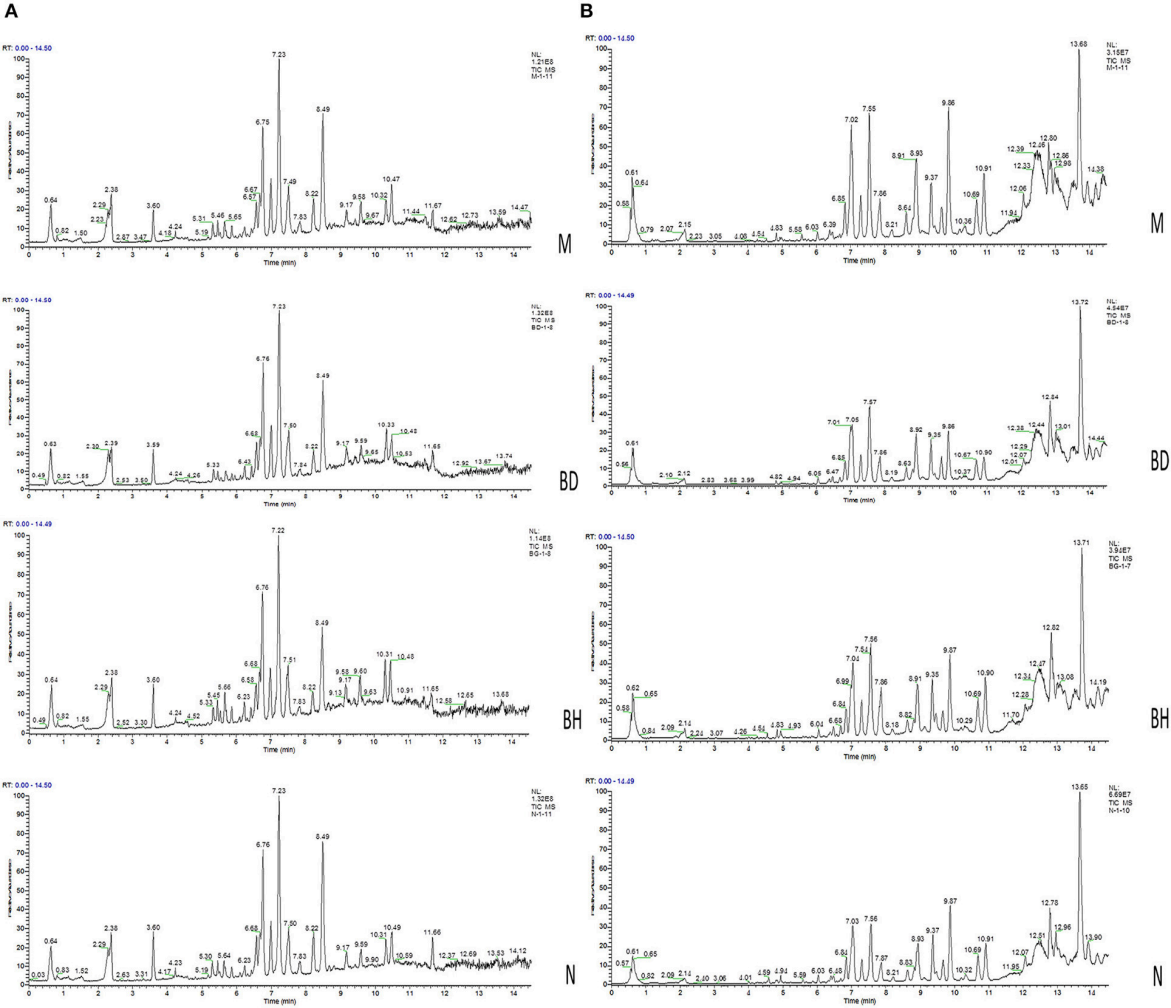
Analysis of the metabolic profiles based on the UPLC/MS spectra of serum samples. Serum BPI chromatograms of APP/PS1 and wild-type mice collected in positive ion mode **(A)** and negative ion mode **(B)**. N denotes the control group, M represents the APP/PS1 transgenic group, BD denotes the 50 mg/kg OMO-treated group, and BH designates the 100 mg/kg OMO-treated group.

**Figure 6:**
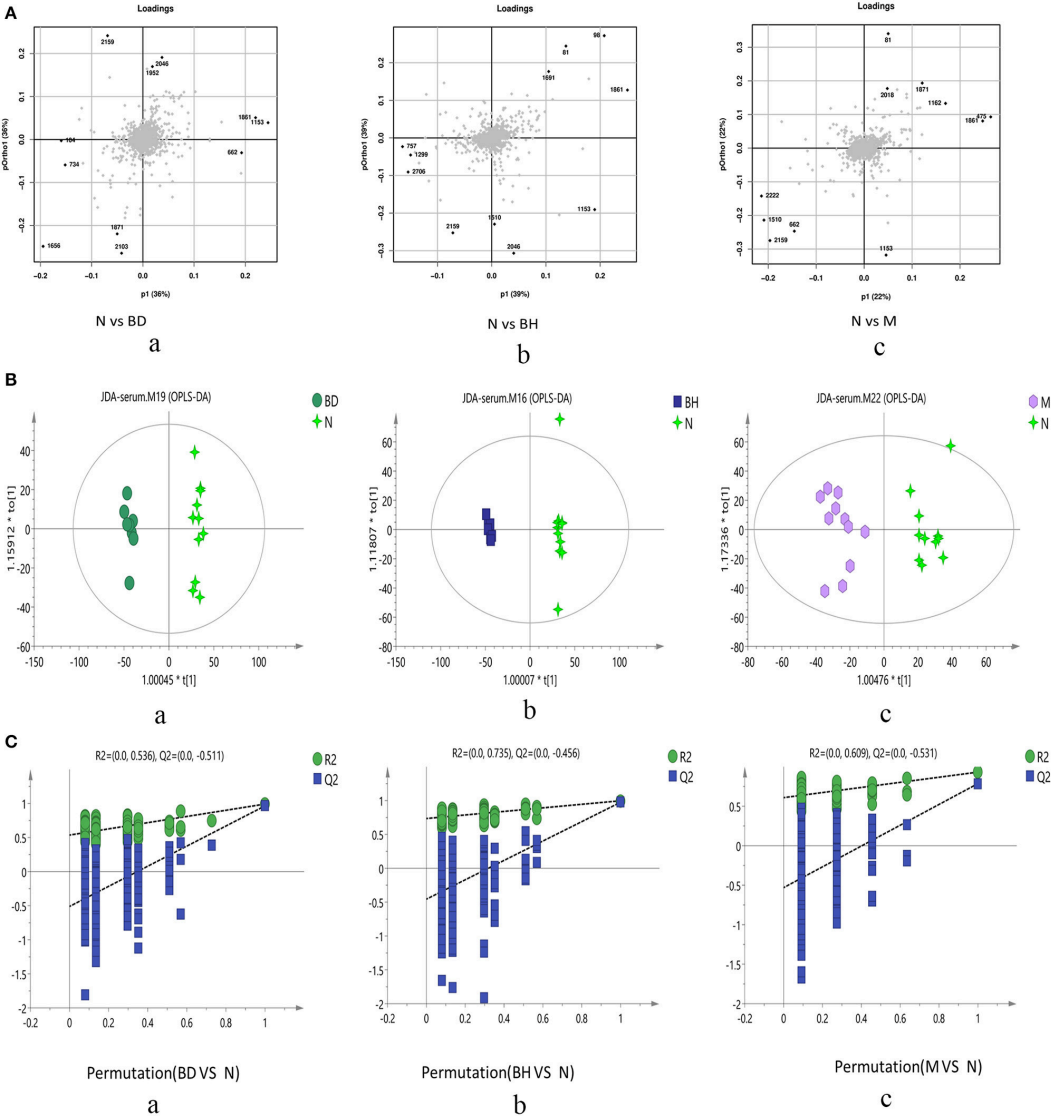
Score plots of metabolite levels in a serum sample. OPLS-DA score plots of the serum metabolic profile **(A)**: (a) N vs. BD, (b) N vs. BH, and (c) N vs. M. Permutation score plots of serum metabolic profiles **(B)**: (a) N vs. BD, (b) N vs. BH, and (c) N vs. M. Loading score plot of the serum metabolic profile **(C)**: (a) N vs. BD, (b) N vs. BH, and (c) N vs. M. N denotes the control group, M represents the APP/PS1 transgenic group, BD denotes the 50 mg/kg OMO-treated group, and BH represents the 100 mg/kg OMO-treated group.

Typical negative and positive base peak APP/PS1 animals are displayed in Figures 5A,B, respectively. All the groups resembled one another in blood patterns of BPI chromatograms, with the exception of a few peaks. Multivariate statistical analysis was conducted with the aim of performing a more thorough investigation of the differences between complicated matrices. The loading plot showed a trend toward separation between any two groups (Figure 6A). Variables with higher loadings (positive and negative) exhibit greater contributions to the differences between the two groups of samples. The labeled metabolites may be relevant to the search for AD biomarkers. A supervised OPLS-DA was utilized to categorize the specimens into 2 blocks, aiming to differentiate between the two kinds of mice. Serum specimens from the two mouse strains were thoroughly isolated according to differences in metabolic patterns using the OPLS-DA loading plot assay. Furthermore, the categorized models were confirmed with the response permutation test (RPT) (38). A permutation plot assisted in the risk evaluation of obtaining a false result from the OPLS-DA. Every blue Q2 dot on the left side was elevated in comparison with primary dots on the right side, suggesting that the primary model was reliable and responsible for the differences observed between the two mouse strains (Figure 6C).

#### Identification of candidate metabolites

**Figure 7:**
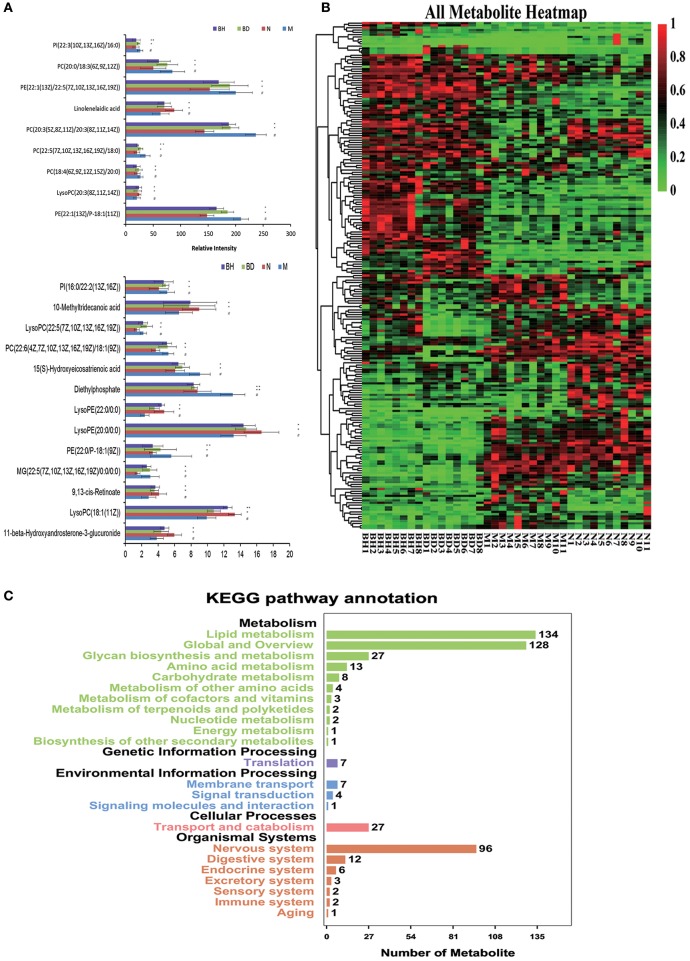
Comparison of the relative intensities of the potential biomarkers **(A)**. KEGG pathway annotations of differentially expressed genes in serum samples **(B)**. Heatmap showing the levels of 24 different metabolites **(C)**. The degree of change is marked with different colors: red represents upregulation, and green indicates downregulation. Each row represents an individual sample, and each column represents a metabolite. N denotes the C57 mice, M represents the APP/PS1 transgenic mice, BD indicates the group treated with 50 mg/kg OMO, and BH denotes the group treated with 100 mg/kg OMO. Values are presented as the means ± SDs of six independent experiments. ^#^*p* < 0.05 compared with the control group; ^*^p < 0.05 and ^**^p < 0.01 compared with the M group.

UHPLC-LTQ was used to evaluate metabolic pathways with the aim of investigating promising pathways that were influenced by APP/PS1 genes. The candidate metabolic products were identified from the MS/MS fragment data and subsequent searches of web-based databases, including KEGG, METLIN and HMDB. Twenty-two candidate markers were initially identified and featured in this way, a summary of which is displayed in Figure 7A (VIP > 1 and *p* < 0.05). We compared the relative intensity of the markers among the APP/PS1 mice, the C57 mice, and the low- and high-dose OMO-treated mice to determine the degree to which those candidate markers were altered. Fourteen metabolites were upregulated, including PI (22:3(10Z, 13Z, 16Z)/16:0), linolelaidic acid, PI (16:0/22:2(13Z, 16Z)), PE (22:1(13Z)/22:5(7Z, 10Z, 13Z, 16Z, 19Z)), PC (18:4(6Z, 9Z, 12Z, 15Z)/20:0), PE (22:1(13Z)/P-18:1(11Z)), 10-methyltridecanoic acid, LysoPC (22:5(7Z, 10Z, 13Z, 16Z, 19Z)), PC (22:6(4Z, 7Z, 10Z, 13Z, 16Z, 19Z)/18:1(9Z)), PE (22:0/P-18:1(9Z)), LysoPE (22:0/0:0), MG (22:5(7Z, 10Z, 13Z, 16Z, 19Z)/0:0/0:0), LysoPC (18:1(11Z)) and 11-β-hydroxyandrosterone-3-glucuronide. The levels of nine metabolites were significantly reduced: PC(20:0/18:3(6Z, 9Z, 12Z)), PC(20:3(5Z, 8Z, 11Z)/20:3(8Z, 11Z, 14Z)), 15(S)-hydroxyeicosatrienoic acid, PC(22:5(7Z, 10Z, 13Z, 16Z, 19Z)/18:0), LysoPC(20:3(8Z, 11Z, 14Z)), diethylphosphate, LysoPE(20:0/0:0) and 9,13-cis-retinoate. Moreover, a clustering analysis of the heatmap of all metabolites revealed the differences in relative levels between the four groups (Figure 7B). Furthermore, we obtained the KEGG pathway annotations of the metabolites (Figure 7C). In the figure, black indicates KEGG primary pathways, and colors represent KEGG secondary pathways; the number represents the counts of differentially produced metabolites in the metabolic pathway.

## Discussion

Pathological changes in AD are associated with microbial infections and gut microbes through four routes of interaction with the brain: direct effects on the vagus nerve, metabolites, direct production or alteration of neurotransmitters, and activation of immune signaling pathways (39). A study in *Nature* (14) also confirmed that the gut microbiota promotes neurodegenerative disease; the gut microbiota are closely associated with neurodegenerative disease in mouse models of Parkinson's disease, and the parkinsonian symptoms are relieved when the gut microbiota is altered. In the present study, OMO markedly modified behavior, regulated neurotransmitter secretion, and ameliorated brain tissue swelling. Moreover, OMO altered the diversity and steady-state composition of the microbial community, and the levels of metabolic products and AD biomarkers were altered. In addition, OMO was an efficacious prebiotic that regulated the composition and metabolism of the gut microbiota in an AD mouse model.

Mechanisms that regulate the GIT microbes have recently been shown to regulate cognition in the host, regardless of whether animals without germs, animals treated with antibiotics or probiotics administration or animals receiving a fecal microbial transplant (FMT) were used (40). The fecal microbial composition was noticeably altered by a 4-day treatment with 50 mg/(kg·day) OMO compared with that of the control group. Our data were consistent with findings from previous studies showing that alterations in the composition of the gut microbiota in APP/PS1 transgenic mice include a reduction in levels of *Bacteroidetes* and an increase in levels of *Firmicutes* (*p* < 0.05), while the OMO treatment reversed those changes (*p* < 0.05), as shown in Figure 5 (Graphian), Figure 6 (OUT and NMDS), indicating that GIT microbes are crucial contributors to host defense. Based on these findings, the gut microbiota acts as a barrier to protect the host from intestinal pathogen attacks (41).

Furthermore, through immunohistochemical staining of global brain tissues in the AD model mice, the present study showed that weight, Aβ_1−42_ levels and staining improved to near normal levels (Figure 1C), indicating that OMO might regulate protein expression and signaling pathways in cells through the help of the gut microbiota. However, additional studies are required to extend the current findings on the effects of the microbiota on mice to humans to provide a basis for gut microbiota-based therapeutic applications of OMO.

In addition, the OMO treatment had an impact on the quantities of some typical GIT microbes, such as Lactobacillus, Bifidobacterium, Bacteroides, Lachnospiraceae, Akkermansia, Ruminococcus, Blautia, S24–7, Lachnospiraceae, Bacteroidaceae and Ruminococcaceae, in APP/PS1 transgenic animals (Figures 4A,B). OMO also affected the gut morphology, mucin production, and gut permeability and reduced dysbacteriosis. According to the results of the 16S metagenome analysis, OMO administration altered the balance of Bacteroidetes and Firmicutes and reduced the abundance of a few beneficial bacterial genera, namely, Akkermansia, Bacteroides, Roseburia, Bifidobacterium, Lactobacillus, and Desulfovibrio, which corroborated previous reports of their roles in metabolic deregulation (42–44).

Importantly, emerging studies have confirmed pathogenic links between GIT microbes and various disorders, particularly the noticeable alteration in quantities of typical microbes in these disorders (45–51). Thus, OMO influences several microbes that affect the generation and secretion of some neurotransmitters and neuromodulators. Murine models of chronic stress displayed similar effects, in which the examined activities, genetic, neuroendocrine and neurochemical alterations observed after the administration of a prebiotic supplement (oligosaccharides and galacto-oligosaccharides) seemed to be regulated in part by short-chain fatty acids (52). Consequently, the therapeutic effects of OMO may be partially attributed to modulatory effects on GIT microbes.

The changes in behavior and the gut microbiota after the administration of OMO coincided with changes in gene expression in blood samples collected from the whole body. A metabolomic analysis of whole-body blood revealed significant changes in the expression of some genes (Figure 7).

We have implemented a metabolomic approach using UHPLC-LTQ Orbitrap mass spectrometry to study the metabolites present in mouse serum, with a special emphasis on revealing whole-body metabolic alterations that might indicate early-stage AD and its progression. Based on the results of the enrichment analysis, lipid metabolites might be related to AD (53). In the category of lipid metabolism, *glycerophospholipid metabolism* and α*-linolenic acid metabolism pathway* were prominently altered in AD. Lipid metabolism is affected by levels of glycerol, propylene glycol, and fatty acid compounds in AD (54). These findings were supported by previous studies reporting elevated levels of polyunsaturated fatty acids in the brain (55, 56). Those fatty acids cause lipid peroxidation and promote the generation of toxic products in response to oxidative stress, resulting in neural damage and promoting AD development.

In the present study, levels of endogenous metabolic products in murine blood differed among the normal, model and OMO-treated groups. These alterations correlate with metabolic diseases, such as monoaminergic neurotransmitters in the nervous system (NS) (57). Thus, blood metabolites represent primary predictors of AD generation.

Pathway enrichment analyses in the metabolomics study indicated that the KEGG pathways *Glutamatergic synapse, Calcium signaling pathway, GnRH signaling pathway, Long-term potentiation, Circadian entrainment*, and *Oxytocin signaling pathway* displayed the greatest alterations during disease pathogenesis. These pathways share mutual hub metabolites, such as pyruvate and oxaloacetate, which are the main compounds required for the TCA cycle, gluconeogenesis, and glyoxylate and dicarboxylate metabolism (58). Pyruvate and pyruvate-oxaloacetate protect NS metabolism by promoting brain-to-blood glutamate efflux (59–61).

Although our understanding of the complicated relationship networks between GIT microbes and the brain is insufficient, prebiotics are known to forcefully regulate the microbial ecology. The influences of the microbial composition, abundance, diversity and functions of all GIT microbes must be examined since they are associated with brain function. Data from our study offer stronger proof of the defensive effect of OMO (which contains prebiotics) and help characterize its effects on the microbiota-brain-gut axis in AD. Using the promising natural chemical product OMO, we were able to identify new therapeutic targets for AD and provide a basis for the further study of AD pathogenesis.

## Author contributions

YX, CD, YJ, LH, LG, SO, ZC, ZJ, and XY conceived and esigned the experiments. YX, CD, YJ, TX, and ZC performed the experiments. YX, CD, LT, HG, and ZJ analyzed the data. YX and CD wrote the paper and edited the manuscript. All the authors read and approved the final manuscript.

### Conflict of interest statement

The authors declare that the research was conducted in the absence of any commercial or financial relationships that could be construed as a potential conflict of interest.
