# Correction: Effects of oligosaccharides from *Morinda officinalis* on gut microbiota and metabolome of APP/PS1 transgenic mice

**DOI:** 10.3389/fneur.2026.1847851

**Published:** 2026-06-03

**Authors:** Yang Xin, Chen Diling, Yang Jian, Liu Ting, Hu Guoyan, Liang Hualun, Tang Xiaocui, Lai Guoxiao, Shuai Ou, Zheng Chaoqun, Zhao Jun, Xie Yizhen

**Affiliations:** 1 Department of Pharmacy, The Fifth Affiliated Hospital of Guangzhou Medical University, Guangzhou, China; 2 The Fifth Clinical School of Guangzhou Medical University, Guangzhou, China; 3 State Key Laboratory of Applied Microbiology Southern China, Guangdong Provincial Key Laboratory of Microbial Culture Collection and Application, Guangdong Open Laboratory of Applied Microbiology, Guangdong Institute of Microbiology, Guangzhou, China; 4 Department of Pharmacy, The Second Clinical Medical College of Guangzhou University of Chinese Medicine, Guangzhou, China; 5 College of Pharmacy, Guangxi University of Traditional Chinese Medicine, Nanning, China; 6 Department of Obstetrics, Guangdong Women and Children Hospital, Guangzhou, China

**Keywords:** oligosaccharides of *Morinda officinalis*, Alzheimer's disease, gut microbiota, metabolomics, APP/PS1 transgenic mice, metabolites

In the published article, there was an error in Figure 1. The Nissl saining of the Model brain slice was inadvertently repeated and also shown for the Nissl saining of BH mouse. The corrected Figure 1 appears below.

**Figure 1:**
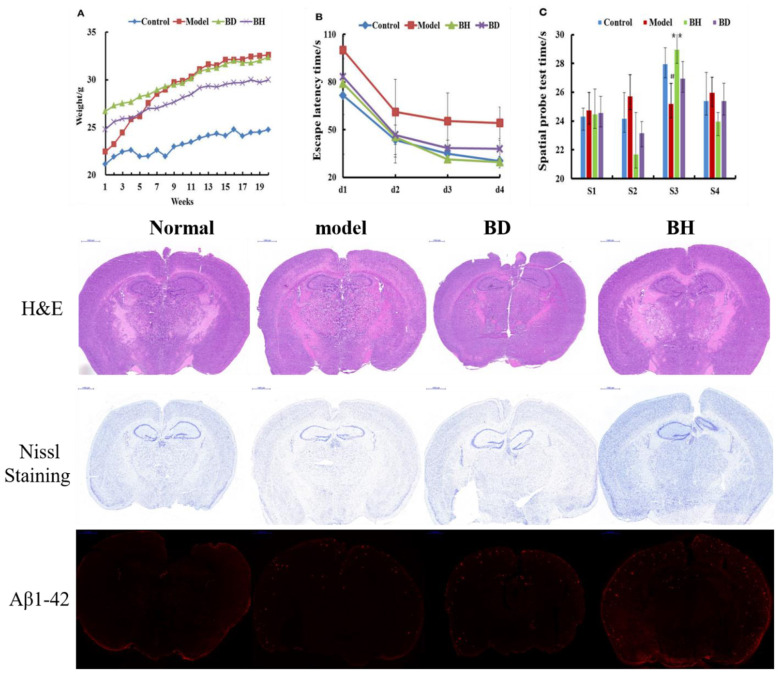
Effect of OMO on APP/PS1 transgenic mice. **(A)** Body weight changes were measured weekly. **(B)** Escape latencies in the H maze and **(C)** probe test results. **(D)** Histopathological changes in brain tissues are shown. N represents the C57 group, M denotes the APP/PS1 transgenic group, BD indicates the group treated with 50 mg/kg OMO, and BH designates the group treated with 100 mg/kg OMO; the treatments were administered for 6 months (*n* = 10). Values are presented as the means ± SDs of six independent experiments. ^#^*p* < 0.05 compared with the control group; **p < 0.01 compared with the model group.

The original article has been updated.

